# Books and Authors

**Published:** 1906-02

**Authors:** 


### BOOKS AND AUTHORS.

---

**Progressive Medicine, Vol. IV,** December, 1905. A quarterly digest of Advances, Discoveries and Improvements in the Medical and Surgical sciences. Edited by **Hobart Amory Hare,** M.D., Professor of Theraputics and Materia Medica in the Jefferson Medical College of Philadelphia. Octavo, 367 pages, 41 engravings, and 5 full-page colored plates. Per annum, in four cloth-bound volumes, $9.00; in paper binding, $6.00; carriage paid to any address. Lea Brothers & Co., Publishers, Philadelphia and New York.

This number of this periodical issue of excellent literature contains contributions on the following topics: treatment, six articles; medicine, five articles; surgery, four articles; neurology, two articles; dermatology and syphilis, one article, two articles; ophthalmology, two articles; and pathology, one article. The contributors have presented an exceptionally interesting group of subjects for consideration, in their several lectures or special articles, which makes the book most instructive, the large number of illustrations contributing in a large measure to this end. Without intent to discriminate with partiality, we invite attention to Dr. Johnston's article on radiotherapy, which is illustrated with 53 engravings, dealing principally with skin diseases.

---

**International Clinics.** A Quarterly of Illustrated Clinical Lectures and especially prepared articles on Treatment, Medicine, Surgery, Neurology, Pediatrics, Obstetrics, Gynecology, Orthopedics, Pathology, Dermatology, Opthamology, Otology, Rhinology, Laryngology, Hygiene and other topics of interest to students and practitioners. By leading members of the medical profession throughout the world. Edited by **A. O. J. Kelly,** A.M., M.D., Philadelphia. Volume III, Fifteenth series. 1905. Philadelphia and London: J. B. Lippincott Co. (Cloth, $2.00)

The contents of the fourth volume for 1905 comprises the following topics and authors: diseases of the digestive tract and allied organs,—the liver, pancreas, and periosteum,—by J. Dutton Steele; genitourinary diseases, by William T. Belfield; diseases of the kidneys, by John Rose Bradford; anesthetics, fractures, dislocations, amputations, surgery of the extremities, and orthopedics, by Joseph C. Bloodgood; and a practical theraputic referendum, by H. R. M. Laudis. The latter contains, among other interesting material, the digest of an article on bathing, by Marvel, (Atlantic City) which embodies the practical observations of large experience. The entire number is one of great value to the busy physician.

---

**Transactions of the Medical Society of the State of New York,** for the year 1905. (Ninty-ninth annual meeting). Edited by **Frederick C. Curtis,** M.D., Secretary. Published by the society. Brander Publishing Company, Fort Orange Press, Albany. 1905.

This being the last volume of transactions the society will issue, The House of Delegates having voted to publish a Journal in which the proceedings and papers read at the meetings will be printed, we need say but little regarding this particular book. We do, however, wish to invite attention to the faithful services of the editor, who is likewise the secretary of the Society, and who has superintended the publication of these volumes for fifteen years. That the work has been well done, no one who has received them will dispute, but comparatively few are familiar with the details of properly preparing the material for the press, and of the labor in seeing it through the printing machine. Only such can fully appreciate how much anxiety and care attends the process of proof reading, of properly locating the illustrations, of the thousand and one small things that need a skilful hand and an artistic eye—but about the book itself; we almost forgot to say that it is quite up to the sandard set by the Society and maintained for many years. The symposia on cerebro-spinal meningitis, and on prostatism, are of a high order of merit and will stand as important features of the ninty-ninth annual meeting.

---

**A Textbook of the Practice of Medicine.** By **James M. Anders,** M.D., Ph.D., LL.D., professor of medicines and clinical medicine at the Medico-Chirurgical College, Philadelphia. Seventh edition, revised and enlarged. Octavo, 1297 pages, illustrated. Philadelphia and London: W. B. Saunders & Co. 1905. (Price: Cloth, $5.50, half-morocco, $6.50 net).

Seven editions in eight years of a treatise on practice, is ample testimony as to the value and popularity of such a work. Anders is an industrious, able teacher, and has the gift of presenting his clinical facts as well as his general knowledge of internal medicine in a forceful, convincing manner. He has availed himself of the opportunities presented by the rapid exhaustion of the several editions of his work, in revising each, and especially is this last one rendered so complete as to make it an oracle of the immediate present on all topics with which he deals.

The treatise is particularly strong as to diagnosis,—inductive and differential,—as well as treatment. The author, in his preface, invites attention to the fact that Rocky Mountain spotted fever, examination of patients for diagnosis of diseases of the stomach, splanchnoptosis, Cammidge's test for glycerose in the urine, and myasthenia gravis, are among the new subjects introduced, while many have been rewritten,—the whole volume having been closely scrutinised, and carefully revised from begining to end. We are pleased to reaffirm our formerly expressed opinion of the work, and to state that it continues to hold a foremost place among American textbooks on the practice of medicine.

